# Cerebral oxygenation measurements during immediate neonatal transition in the delivery room: a systematic review

**DOI:** 10.1038/s41390-025-04084-z

**Published:** 2025-05-12

**Authors:** Rania Selim, Arangan Kirubakaran, Jay Banerjee

**Affiliations:** 1 Institution: Faculty of Medicine, Imperial College London, London, UK; 2 Institution: Department of Neonatology, Imperial College Healthcare NHS Trust, London, UK

## Abstract

**Objective:**

To systematically review the use of NIRS measured cerebral oxygenation and analyse these parameters during the immediate postnatal period.

**Data Sources:**

EMBASE, MEDLINE, and Maternity and Infant Care databases using keywords: “Infants,” “NIRS,” and “Cerebral oxygenation.”

**Study selection:**

Inclusion criteria were clinical trials and observational studies measuring cerebral oxygenation up to 15 min of life. Exclusion criteria were non-human studies, non-English articles and case reports.

**Data extraction:**

Two authors independently performed study selection, data extraction, and risk of bias assessment. Cerebral regional tissue Oxygenation (CrSO_2_) and cerebral fractional tissue oxygenation extraction (cFTOE) values were extracted.

**Results:**

Fifty nine studies, out of 4067 were included in the qualitative analysis. Studies included aimed to establish oxygenation reference ranges, assess the impact of delivery mode, cord clamping, and delivery room interventions on cerebral oxygenation, and evaluated its role in predicting long-term neurodevelopmental outcomes. Most studies focused on term neonates experiencing normal neonatal transitions. Aggregate mean values for CrSO_2_ and cFTOE in the first 15 min of life were calculated, showing that a steady state is achieved by 10–15 min of life. ANOVA demonstrated no significant differences between preterm and term infants in CrSO_2_ (*p* = 0.54) and cFTOE (*p* = 0.50).

**Conclusions:**

NIRS measurement of CrSO_2_ is feasible and can be used alongside other clinical tools to inform delivery room management. There were no significant differences in CrSO_2_ or cFTOE between term and preterm infants although most studies focussed on late preterm infants. Future research is therefore required for extremely preterm infants, those requiring ventilatory management, or those with congenital anomalies.

**Impact:**

- There is a knowledge gap regarding cerebral oxygenation patterns during immediate neonatal transition. NIRS can be used to monitor and guide clinical management in delivery room, helping to inform clinicians about cerebral oxygenation during the transition.
- This study provides a comprehensive review of NIRS applications in measuring neonatal CrSO_2_ up to 15 min after birth, producing a collated reference range graph with no significant differences found between gestations.
- This study enhances the understanding and application of NIRS during the immediate transitional period, providing insights that can improve delivery room management practices and guide interventions for both term and preterm infants.

## Background

The newborn transition involves a rapid series of complex physiological changes enabling a successful adaptation to extra-uterine life, including the clearance of fetal lung fluid, decreases in pulmonary vascular resistance, catecholamine release and surges in cortisol.^1^ This is often impaired in preterm birth or Caesarean sections.

Whilst heart rate and peripheral oxygen saturation levels are routinely monitored during resuscitation, relying solely on these metrics has its limitations.^2–4^ It is logical to assume that infants may benefit from an individualised approach based on feedback from physiological parameters, based on their unique risk profiles relating to gestational age, birthweight, maternal comorbidities and antenatal steroid use.

Opportunities for additional monitoring during the transition can therefore further enhance neonatal care, such as in cord management. The well-documented benefits of deferred cord clamping in preterms^5,6^ has led to the introduction of optimum cord management in all infants, involving maintaining an open umbilical cord for at least 60 seconds,^7^ although the exact timing for its optimum benefit remains unclear.^8^

Near-infrared spectroscopy (NIRS) is a non-invasive monitoring tool to assess cerebral tissue oxygenation, expressed variably as cerebral regional saturation percentage (CrSO_2_) and cerebral tissue oxygenation index (cTOI) depending on the manufacturer, and when measured alongside SpO_2_, calculates fractional tissue oxygen extraction (FTOE).^9^

This review evaluated the current evidence examining NIRS-measured cerebral oxygenation in the immediate transition period in infants in the delivery room, its potential use and its limitations. We also aimed to analyse the data and describe trends for different cerebral oxygenation parameters in the first 15 min after birth.

## Materials & Methods

This systematic review was conducted following the Cochrane guidelines for Systematic reviews and registered on PROSPERO (Registration number: CRD42023470629) and followed guidelines from the Preferred Reporting of Items for Systematic Reviews and Meta-Analyses (PRISMA) statement (Supplementary Material 1).

### Search strategy

EMBASE, MEDLINE and Maternity and Infant care databases were searched systematically with help of a sciences librarian (RJ). The search included the following components: Infants, NIRS, Cerebral Oxygenation (Supplementary Material 2). The initial search was performed in September 2023 to October 2023, with an updated search from February to March 2025.

All clinical trials and observational studies published in English measuring cerebral oxygenation in the immediate cerebral oxygenation period (up to 15 min of life) within the delivery room were included in this review. Exclusion criteria included, non-human studies and case reports.

### Data extraction and synthesis

Covidence systematic review software (Veritas Health Innovation, Melbourne, Australia) was used for study selection and data extraction. Duplicates were removed and two reviewers (R.S, A.K.) independently performed title and abstract screening for eligibility, with conflicts resolved by a third reviewer (J.B.). Articles included in this study are related to cerebral oxygenation measurement using NIRS in the first 15 min of life. Data extraction included: 1) Publication identification details, 2) Study design, Population, Participant Gestational Ages and Participant exclusion criteria, 3) NIRS machine model utilised in the study, 4) Use of NIRS, 5) Duration of cord clamping 6) Outcomes measured in the study. Data on graphs was extracted using WebPlotDigitizer 4.8,^10^ and mean values were estimated if median or centile values were given. Aggregate mean values were weighted based on sample size prior to using the ANOVA one-factor test.

Study quality and applicability were assessed independently by two authors (R.S., A.K.) using Critical appraisal skills programme checklist for Prospective Cohort studies (CASP).^11^

## Results

**Fig. 1:**
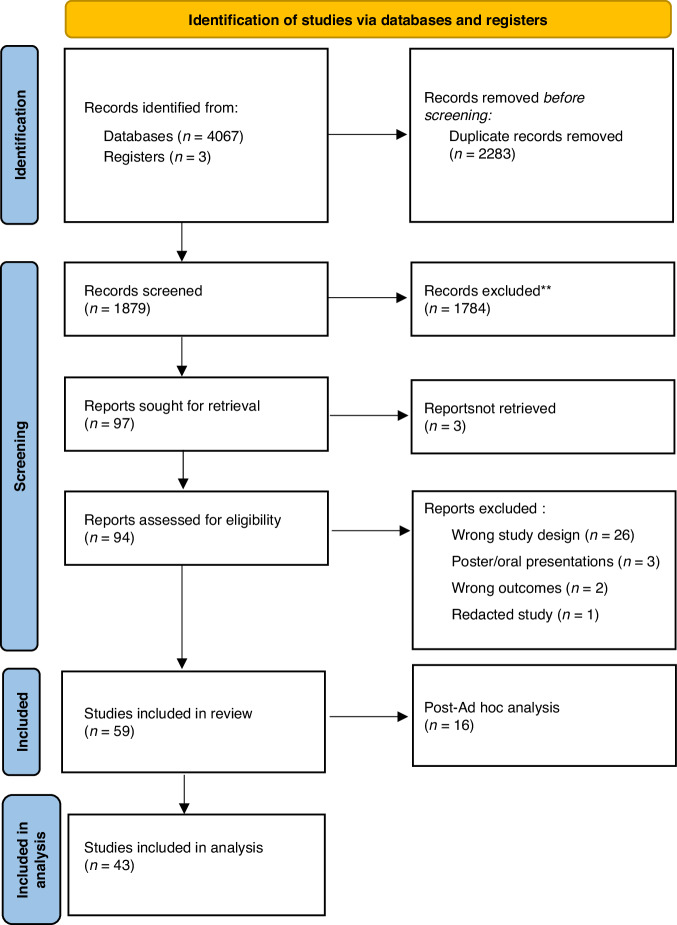
PRISMA (Preferred Reporting Items for Systematic Reviews and Meta-Analyses) flow diagram.

**Fig. 2:**
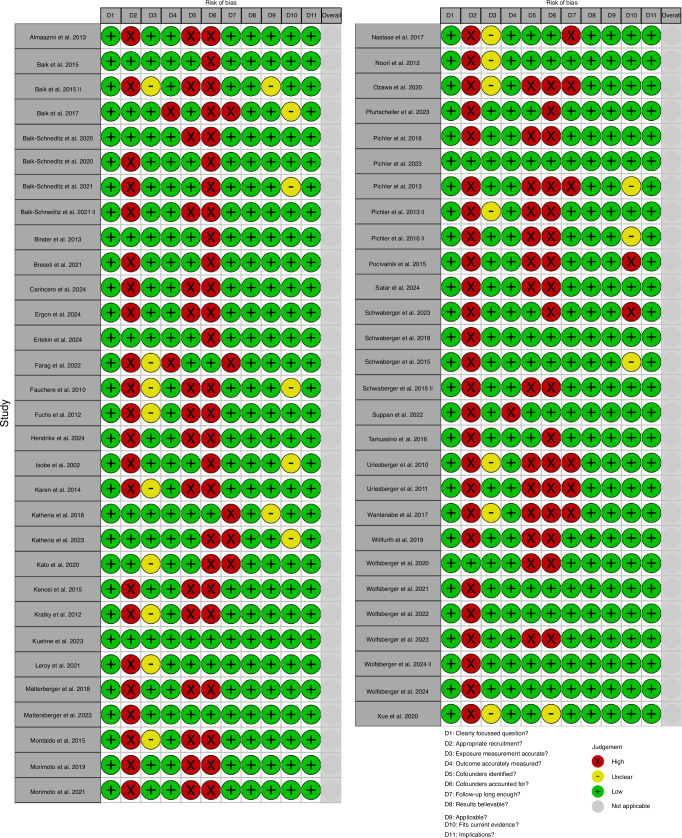
Quality Assessment table representing risk of bias in different domains of Critical Appraisal Skills Programme Checklist.^11^

From the literature search, 1879 abstracts were identified of which 59 studies were reviewed for inclusion. Of these, 16 studies were retrospective post-hoc analyses which were included in the qualitative analysis and reviewed for discussion only. Hence, a total of 43 prospective studies including 4111 patients were included for final statistical analysis (Fig. 1). Detailed characteristics of each study are summarised in Table 1. Figure 2 shows the quality assessment using a CASP prospective cohort study checklist.^11^

**Table 1:** Studies using NIRS during the neonatal transition.

Study	Population and Design	NIRS machine	Outcomes Measured	Duration of Cord Clamping	Findings	Limitations
*Mode of Delivery Comparisons*						
Almaazmi et al. ^37^	Prospective observational study of 46 healthy term infants born via CS, SVD and forceps delivery.	FORE-SIGHT	cStO_2_ and FTOE during first 10 minutes after delivery, comparing healthy to previously published VLBW infant cohort.	Not documented	Median (IQR) cStO_2_ at 2 minutes for SVD (n = 15): 42% (39-46), CS (n = 17) 42% (31-52)	Low number of instrumental deliveries (n = 4).
					Instrumental (n = 4) 36% (20-53); at 8 minutes median cStO_2_: 73% (62-77).	
	Exclusion: positive pressure ventilation, additional oxygen or congenital malformations.					
	GA: 39.9 (39.6-40.6) SVD, 39.0 (38.9-39.1) CS, 38.0 (37.2-39.4) assisted					
Farag et al. ^14^	Prospective observational study of cerebral and peripheral oxygen saturations in 60 healthy term neonates born via SVD and CS.	INVOS 5100 C	Spot CrSO_2_ checks at 1, 5, 10 minutes after birth.	Not documented	Mean CrSO_2_ values: 42% at 1 m, 71% at 5 m, 81% at 10 m.	Instrumental deliveries, babies needing respiratory support or born premature excluded.
					Mean cFTOE values: 0.44 at 1 m, 0.21 at 5 m, 0.15 at 10 m.	
	Exclusion: IUGR, perinatal asphyxia, instrumental deliveries				Mode of delivery was the most significant factor affecting NIRS-measured CrSO_2_.	
	GA: 39.0 (38.0-41.0)					
Isobe et al. ^40^	Prospective observational study comparing the effect of vaginal delivery or elective CS on cerebral haemoglobin oxygen saturation in 26 term neonates.	IMUC-7000	SbO_2_ and SaO_2_ measured at 10 second intervals during the first 15 minutes of life.	Not documented	Standard delivery group: 29 ± 17% (2 m), 68 ± 6% (8.5 m), 66 ± 7% (15 m)	Unequal sample size (n = 6 for CS).
					CS: 40 ± 11% (2 m), 68 ± 4% (8.5 m) but fell to 57 ± 5% (15 m; statistically significant)	
	GA: 37-41					
Karen et al. ^39^	Prospective observation study assessing the impact of vacuum-assisted delivery on cerebral oxygenation in 15 neonates compared to results from Fauchere et al.	NIRO 300	O_2_Hb, HHb, TOI, THI measured from birth to first 15 minutes of life.	Not documented	TOI was 11.6 ± 6.3% lower in CS (53 + 5%) than vacuum-assisted deliveries (65 + 6%), which was statistically significant.	Small sample size.
	Exclusion: genetic syndrome, congenital malformation					
	GA: 40 (vacuum), 38 (CS)					
Morimoto et al. ^41^	Prospective observation study of 31 healthy term neonates comparing CBV and cerebral haemoglobin oxygen saturation (ScO_2_) between vaginal delivery and elective caesarean section.	TRS system	CBV, ScO_2_	30 seconds	CBV was significantly higher in the VD group compared to the CS group during the first 4 minutes after delivery. CVB decreased in both groups during the subsequent 15 minutes.	Well term babies included only. Instrumental deliveries not included.
	Exclusion: need for respiratory support, hospitalisation due to hypoglycaemia or infection; emergency caesarean section because of obstructed labour and foetal distress; and abnormal optical properties.					
	GA: 39.7 ± 1 (VD) and 38.5 ± 1 (CS)					
Urlesberger et al. ^38^	A prospective, observational study of 63 healthy term infants comparing cerebral oxygenation between vaginal deliveries or Caesarean section.	INVOS	rSO_2_, SpO_2_, Arterial oxygen saturation, HR, BP for the first 10 minutes after delivery	Not documented	There was no statistically significant difference between groups in rSO_2_ brain between caesarean delivery infants and vaginally delivered infants.	Only infants born via CS and VD included.
	Exclusion: Congenital malformations, instrumental deliveries, need for respiratory support/inspired oxygen					
	GA: 38.5 ± 1.1 (CS) and 40 ± 1.3 (NVD)					
Willfurth et al.*^42^	Post-hoc analysis assessing the impact of maternal anaesthesia on the cerebral regional oxygen saturation of 118 neonates during immediate transition following Caesarean section.	INVOS 5100	CrSO_2_, SpO_2_, HR, cFTOE measured during the first 15 mins of life.	30 seconds	There were no statistically significant differences in CrSO_2_ and cFTOE in the GA group compared to the SA group in both term and preterm.	Small sample size. Indication for delivery not recorded.
	GA: term 38.8 ± 0.9, preterm 32.0 ± 2.9				cFTOE values were similar in both groups for term and preterm neonates.	
	Exclusion criteria: Missing measurements or incomplete demographic data, those not born via CS					
					FiO_2_ was found to be significantly higher in GA neonates in both term and preterm neonates.	
*Healthy Term Infants Only*						
Baik et al. ^19^	Prospective observational study measuring cFTOE and cTOI in 140 term babies after birth to establish reference ranges.	NIRO 200NX	Continuous cFTOE and cTOI measurement for 15 minutes after birth.	30 seconds	50^th^ Centile (10^th^ to 90^th^) values:	No delayed cord clamping. Preterm infants or babies needing respiratory support excluded.
					cTOI:	
	Exclusion: SVD/instrumental delivery, need for respiratory support, preterm				56% (39-75) at 2 m, 66% (50-78) at 5 m, 75% (62-85) at 10 m, 73% (61-83) at 15 m	
					cFTOE:	
	GA: 38.8 ± 0.9				0.24 (0.11-0.44) at 2 m, 0.2 (0.1-0.35) at 5 m, 0.21 (0.09-0.35) at 10 m, 0.24 (0.13-0.37) at 15 m	
Baik-Schneditz et al. ^21^	Prospective observational study evaluating differences in cardiac output and sex in 99 term neonates.	NIRO 200NX	Measured HR, cTOI, cardiac output and SpO_2_ at 5, 10 and 15 minutes of life.	30 seconds	No significant differences between cTOI but cardiac output was significantly higher in male babies at 15 minutes of life.	Other methods of birth excluded.
					cTOI:	Need for medical support was also an exclusion criteria.
	Exclusion: SVD/instrumental deliveries, need for medical support, transition lasting >15 minutes, congenital malformations.				Male: 63.4% (60.8-66.1) at 5 m, 73.1% (70.3-75.9) at 10 m, 72.5% (69.7-75.3) at 15 m	
					Female: 63.4% (60.8-66.1) at 5 m, 73.2% (70.6-75.7) at 10 m, 73.6% (71.0-76.1) at 15 m	
	GA: 39.0 (male), 38.8 (female)					
Baik-Schneditz et al. ^24^	Prospective Observational Study of 99 term neonates born via CS to assess correlation between Cardiac Output and cTOI.	NIRO	CO, HR, SpO2, cTOI	30 seconds	No significant correlation between CO and cTOI was found.	Only term neonates born via CS included.
					cTOI showed statistically significant increase from min 10 to min 15.	
	GA: 38.8 (38.3-39.3)					
Carnicero et al. ^20^	Prospective observational study investigating CrSO_2_ and CFTOE measurements in 44 healthy term babies born via CS in the immediate transition period.	MASIMO ROOT	CrSO_2_SpO_2_, cFTOE, Apgar scores, HR	Not documented	CrSO_2_ with MASIMO increased from 2 min to 8 min before plateauing, rising to 83% at 10 min. CFTOE was initially high at 0.25 and then plateaued to 0.19 at 15 min. There were significant differences in CrSO_2_ when compared to NIRO measurements from 3 mins and from 6 mins upwards in cFTOE measurements.	Healthy neonates born via CS included only. Small sample size.
	Exclusion: SVD, Apgar score <8 at any time, preterm, neonates needing respiratory support or supplementary oxygen, congenital malformations, genetic syndromes, neurological or cardiac disease or those without continuous signals					
	GA: 39.7 ± 1.67					
Ergon et al. ^32^	Prospective observational study investigating the effect of body position on CrSO_2_ and cFTOE in 60 term neonates.	INVOS 5100 C	CrSO_2_, cFTOE, HR, SpO_2_ measured for 10 minutes after birth.	Not documented	No significant differences between positions and HR, CrSO_2_, cFTOE or SpO_2._	Elective Caesarean section only. Other methods of birth excluded. Blood pressure not measured.
	Exclusion: SVD, preterm, congenital malformations, APGAR < 7, respiratory support				R frontal: supine 72.5% (5 m) & 82.8% (10 m), prone 77.3% (5 m) & 82.3% (10 m), right side 72.5% (5 m) & 80.2% (10 m), left side 73.3% (5 m) & 82.3% (10 m).	
	GA: 39				L frontal: supine 72.9% (5 m) & 83.2% (10 m), prone 76.4% & 83.0% (10 m), right side 75.7% (5 m) & 82.5% (10 m), left side 74.5% (5 m) & 83.5% (10 m)	
Fauchere et al. ^23^	Prospective observational study of changes in oxygenated/deoxygenated haemoglobin and tissue oxygenation index in 20 term neonates born via elective Caesarean section.	NIRO 300	TOI measured from birth to 15 minutes of life.	Not documented	Changes in O_2_Hb (3.4µmol/L), TOI (4.2%/min), SpO_2_ (4.6%/min), HHb (-4.8µmol/L) reached a plateau within approximately 8 minutes and were statistically significant.	Elective Caesarean section only.
	Exclusion: need for resuscitation, congenital malformation					
	GA: 38 + 1 (36 + 6-40 + 2)					
Kato et al. ^15^	Prospective observational study to develop reference ranges of brain regional oxygen saturation in 100 term babies.	KN-15	SpO_2_, rSO_2_ measured at 1, 3,5 and 10 minutes of life.	Not documented	Mean rSO_2_ at 1 minute: 43%.	Measurement of KN-15 is shallower than conventional NIRS.
					rSO_2_ at 10 minutes: 56%	No preterm babies or birth asphyxia included.
	Exclusion: congenital anomalies, multiple pregnancy, meconium-stained liquor/olighydramnios, asphyxia				Plateau at around 60% between 8-10 minutes of life.	
	GA: 37.9 ± 1.2					
Leroy et al. ^74^	Exploratory pilot study of cerebral oxygenation in 20 term neonates.	NIRO 200NX	TOI, cerebral tissue oxyhaemoglobin (HbO), tissue deoxyhaemoglobin (HbR) and cerebral blood volume (HbT) measured in first 10 minutes of life	Not documented	TOI trajectory for all newborns followed a rapid initial increase (phase 1) and a plateau (phase 2) within 300 seconds. Rapid increase from onset to 244.2 s was significant.	Small sample size. Temperature and glucose not evaluated.
	Exclusion: respiratory support					
	GA: 39.5 (38.8-40.8)					
Montaldo et al. ^75^	Prospective observational study of 61 term infants delivered by elective CS examining cerebral, renal and mesenteric oxygen saturation for first 9 hours of age.	EQUANOX 7600	CrSO_2_, RrSO_2_, MrSO_2_, RtFOE, MtFOE, CtFOE measured continuously for first 15 minutes of life.	Immediate	Between 3-7 minutes RrSO_2_ and MrSO_2_ were significantly lower than CrSO_2_ with RtFOE and MtFOE being significantly higher than CtFOE at 3-7 minutes of life. RrSO_2_, MrSO_2_ increased more slowly than CrSO_2_ within first 7 minutes of life.	Babies born via CS only. Small sample size. Unbalanced sex distribution. No information about missing data.
	Exclusion: congenital malformations, IUGR, supplemental oxygen, need for resuscitation					
Morimoto et al. ^76^	Prospective observational study of 7 term infants born via SVD/instrumental deliveries.	TRS-21	ScO_2_, cerebral blood volume	30 seconds	Mean ScO_2_ at 2 minutes 48.0%, 53.9% at 3 minutes, 62.5% at 5 minutes, 67.2% at 10 minutes and 64.3% at 15 minutes.	No systemic haemodynamic or blood gas data, small sample size.
	Exclusion: respiratory support, hypoglycaemia/infection, congenital abnormalities				CBV at 2 minutes 3.09 mL/100 g; 3.01 at 3 minutes, 2.69 at 5 minutes, 2.40 at 10 minutes and 2.08 at 15 minutes	
	GA: 38-40					
Nastase et al. ^16^	Prospective observational study of 74 neonates to determine normal ranges for cerebral oxygenation in the first 10 minutes of life.	INVOS 5100 C	cFTOE, rcSO_2_	Not documented	All newborns showed a gradual increase of regional brain saturation (rcSO_2_) parallel to SpO2 from an average of 35.8% (15.0% -62.0%) to 1 minute at 65.3% (46% -85%) at 5 minutes and reaching peak values of 76.8% (66.7% -87.4%) at 10 minutes of life.	No information about missing data and no description of quality control measures.
	Exclusion criteria: respiratory support, suspected or known brain malformations, or birth complications (e.g. vacuum extraction or forceps application).					
	GA: 38 + 3					
Noori et al. ^22^	Prospective observational study of 20 term neonates born via SVD measuring cardiac function, cerebral haemodynamics, oxygen saturation and cerebral oxygenation in first 20 minutes of life.	INVOS	CrSO_2_, preductal SpO_2_ measured in first 20 minutes of life. Echocardiography and Doppler ultrasonography performed during first 20 minutes of life.	Not documented	CrSO_2_ 47 ± 21% at 1 minute of life, 83 ± 9% at 8 minutes and 73 ± 12% at 20 minutes of life. No change in cFTOE seen after early transition.	Cerebral blood flow not measured.
	Exclusion: congenital malformations, SGA/LGA, instrumental deliveries, hydrops					
	GA: 39.1 ± 1.3					
Ozawa et al. ^18^	Prospective observational study of 127 neonates to determine reference ranges for cerebral oxygenation values of KN-15 device.	KN-15	CrSO_2_ values were measured in first 10 minutes of life.	Not documented	Median CrSO_2_ at 1 minute: 44.6% (39-48.3), 2 minutes 46% (41-50.6), 3 minutes 48.3% (44-53.4), 4 minutes 50.7% (46.5-55.8), 5 minutes 52.4% (48.4-56.3), 53.1% (49-56.4), which were all statistically significant when compared to 10 minutes (56.2%). There were no statistically significant differences between SVD or CS.	Small sample size from normal vaginal deliveries (n = 19). No differences observed after 10 minutes.
	Exclusion: supplemental oxygen/ventilation, congenital anomalies, instrumental deliveries, pregnancy complications					
	GA: 37.6 (37-41 weeks)					
Schwaberger et al. ^36^	Observational study of 109 term infants born via elective CS to evaluate cerebral blood volume and cerebral tissue oxygenation index (cTOI) in the first 15 minutes after birth.	NIRO 200-NX	Cerebral Blood flow, SpO2, cTOI, changes in total haemoglobin and HR in the first 15 minutes after birth.	Immediate Cord clamping ( < 30 s)	From 2 to 15 minutes, there was a decrease in HbT (17umol/l) and CBV 1.0 ml/100 g brain.	Impact of unaccounted potential confounders on the analysis of cerebral oxygenation trends over time.
	Exclusion: Infants born vaginally, respiratory support and congenital malformations.				cTOI increased from minutes 2-6 and then plateaued between the values of 70-75%.	
	GA: 38 + 6 weeks					
Tamussino et al. ^31^	A single center prospective observational study analysing low cerebral activity and cerebral regional oxygenation saturation in 59 term neonates during immediate transition after birth.	INVOS	AEEG, CrSO_2_, cTFOE SpO_2_ and HR in the first 15 minutes after birth	Not documented	Study group CrSO_2_:	Small number of infants in study group.
					4 m - 50 ± 20.2, 7 m 63 ± 14.7, 10 m: 67 ± 17.9	
	Exclusion: Congenital malformation, infants born via CS under GA					
					Control group CrSO_2_:	
	GA: term (not documented)				4 m - 59 ± 19.4, 7 m 78 ± 11.3, 10 m: 79 ± 10.7	
					Cerebral regional oxygenation was significantly lower and cFTOE was significantly higher in the study group compared to control group.	
Urlesberger et al. ^34^	A prospective observational study of cerebral oxygenation (rSO_2_)in the first 10 mins of 59 term infants after caesarean delivery.	INVOS 5100	RSO_2_, Arterial Oxygen Saturation, HR, FTOE in the first 10 minutes after birth.	Not documented	Mean rSO_2_ brain increased rapidly to 7 minutes with no further change.	Only infants born via CS were included.
					Cerebral oxygenation increased from 45% at 3 minutes to around 70% at 10 minutes. Fractional tissue oxygen extraction decreased during the first minutes of life, by which values of the brain did not change significantly after 5 minutes.	Blood gas analysis not included
	Exclusion: Congenital malformations, need for respiratory support or additional oxygen.					
	GA: 39 ± 1					
Watanabe et al. ^35^	A prospective observational study of 32 term infants to determine feasibility of measuring cerebral oxygenation immediately after birth.	KN-15	CrSO_2,_ SpO_2_ during the first 10 minutes	Not documented	Median CrSO_2_ and cFTOE significantly increased from 43% in 1 min to 49% in 4 min with no further statistical change.	Small sample size of vaginal births (13%)
	Exclusion: Infants requiring respiratory support or additional inspired oxygen, congenital malformations, instrumental deliveries.					
	GA: 38 ± 1					
Wolfsberger et al.*^26^	Post hoc analysis of Bresesti et al. ^56^ investigating differences in cerebral oxygenation during transition between 12 term paired neonates based on prenatal tobacco exposure.	INVOS 5100 C	CrSO_2_, cFTOE, SpO_2_, HR within 15 mins after birth	30 seconds	From Birth until 5 mins after birth, the smoking group had significantly lower CrSO_2_ and significantly higher cFTOE than no smoking group.	Self-reported smoking data was used.
						No objective measure of tobacco consumption.
	Exclusion criteria: Major congenital anomalies, need for medical support including medications, supplementary oxygen and/or respiratory support including CPAP or intubation.				At 3, 4, and 5 minutes, the values for the smoking group were 41.4, 50.3, and 59.4, while for the non-smoking group, they were 54.7, 62.9, and 73.3, respectively.	Small sample size due to strict matching criteria.
	GA: 39.1 (38.8-39.3) in smoking cohort and 39.1(38.7-39.2) in non-smoking cohort.					
Xue et al. ^30^	Prospective Observational study of cerebral oxygenation during transition in 418 neonates born after caesarean section.	FORE-SIGHT MC-2000	CrSO_2_, SpO_2_, HR between 2 minutes till 60 minutes after birth	Immediate	Cerebral oxygenation of non-oxygen-inhaled intrathecal anaesthesia neonates without medical support 43% of neonates reached a brain oxygen saturation relative stablisation by 7 minutes, 78.7% achieved brain oxygen saturation relative stablisation by 8 minutes.	Small sample size for CS under GA.
					60% of newborns experienced a 20-50 second period between 2 and 5 minutes of life where there was a decrease in cerebral oxygenation (5-30%)	Only infants being born by CS were included.
	Exclusion criteria: Congenital malformations or haematological disease.					All monitoring performed in nursing room
	GA: 37-42 weeks					
*Premature infants Only*						
Baik et al. ^47^	Prospective observational study measuring CrSO_2_ and IVH development in 24 matched preterm infants ( < 32 weeks).	INVOS 5100	Continuous HR, SpO_2_ and CrSO_2_ measurement for 15 minutes after birth. CrUSS at days 4, 7, 14 and pre-discharge.	Early clamping (n = 12) and 60 seconds (n = 12).	Significant differencein CrSO_2_ between IVH and non-IVH groups (p = 0.003), with decreased values between minutes 7-15.	Small sample size.
					IVH grades I-II: 50%, Grade III: 50%	Cerebral blood flow and pCO_2_ not measured.
	Exclusion: congenital malformations					
					Mean ± SD CrSO_2_ at 2 to 15 minutes:	
	GA: 25.7 ± 2.6 (IVH), 26.4 ± 2.1 (non-IVH)				IVH group: 22±11to 52 ± 19%	
					Non-IVH group: 27±11to 73 ± 6%	
Bresesti et al.*^56^	Retrospective analysis of four studies assessing impact of degree of bradycardia and the presence of hypoxemia on crStO2 and cFTOE in 150 preterm infants needing respiratory support.	NIRO 200-NX or INVOS 5100 C	Continuous measurements of HR, FiO_2,_ SpO_2_, cFTOE, crStO_2_ for 15 minutes after birth.	30 seconds	Degree of bradycardia was not significantly associated with crStO_2_ and cFTOE. Degree of bradycardia with additional presence of hypoxaemia ( < 80%), was then associated with crStO2 and cFTOE.	pCO_2_ and blood glucose not measured. No control group for term infants.
	Exclusion: term neonates, those without respiratory support, congenital malformations or missing data.					
	GA: 33 (31-34)					
Fuchs et al. ^12^	Prospective observational study of cerebral tissue oxygen saturation in 51 very low birthweight (VLBW) infants after birth.	FORE-SIGHT	CrSO_2_ and cFTOE measured continuously for first 10 minutes of life.	Not documented	Median StO_2_ was 37% (31-49) at 1 m and reached a steady state of 61-84% at 7 m.	Inaccurate oximeter results during hypoxia and bradycardia. No haemodynamic measurements. No data on cord clamping.
	Exclusion: BW > 1.5 kg					
	GA: 27.8 ± 2.6					
Katheria et al. ^46^	Prospective observation study assessing whether cerebral oxygenation and electroencephalography can predict intraventricular haemorrhage formation in 127 preterm babies.	FORE-SIGHT	StO_2_, SpO_2_, HR, MAP, FiO_2_ and EEG measured for 72 hours from birth. CrUSS performed at 12 & 72 h.	Not documented	Infants who developed severe IVH/death had a lower StO_2_ at 8-10 minutes of life. A StO_2_ threshold of 66% at 7-10 minutes of life had a sensitivity of 89% and specificity of 81% for adverse outcomes.	Small sample size (n = 4) for severe IVH/death.
	Exclusion: congenital anomalies, head bruising					
	GA: 28 ± 2 (non-IVH), 26 ± 2 (IVH)					
Kenosi et al. ^43^	Prospective observational study of 47 preterm infants exploring effects of fractional inspired oxygen delivery on cerebral oxygenation.	INVOS 5100	CrSO_2_ measured for 48 hours from birth. FiO_2_ titrated as per local resuscitation protocol and recorded by independent researcher.	Immediate	The low FiO_2_ group ( < 0.3) had statistically higher rSO_2_ values (+7.1%), but this did not change with time.	Infants only categorised into ‘high’ or ‘low’ FiO_2_ group; questionable validity for babies requiring transient high FiO_2_.
						No quality control conducted over measurements.
	Exclusion: congenital anomalies, infants not expected to survive, >32 weeks gestation					
	GA: 29.4 ± 1.6					
Wolfsberger et al.*^67^	Post-hoc analysis of COSGOD III trial (Pichler) exploring the relationship between cerebral oxygenation monitoring after birth and fidgety movements between 6 to 20 weeks CGA, identified in 171 neonates.	INVOS 5100	CrSO_2_, SpO_2_, FiO_2_, HR measured in the first 15 minutes of life.	Not documented	No significant differences in survival between two groups. NIRS monitoring did not have an impact on fidgety movements or mortality.	Post-hoc analysis. Most babies born via CS. Blood gas parameters not included.
	GA NIRS: 29.4, control: 28.7		Fidgety movements analysed via video recordings between 6-20 weeks CGA.			
Wolfsberger et al.*^51^	Retrospective observational study of cerebral oxygenation within the first 15 minutes of 42 preterm neonates ≤32 weeks and/or ≤1500 g.	INVOS 5100 C	CrSO_2_, cFTOE, SpO_2_ and HR during first 15 minutes after birth, Bayley Scale of Infant Development III at 2 years.	30 seconds	Adverse outcomes group was defined as those preterm neonates who died or survived with severe disability (cognitive Bayley scales of Infant development III ≤ 70 or inability to perform test).	Small sample size.
						Loss of participant follow up in favourable outcome group.
	Exclusion criteria: Neonates with less than 50% cerebral NIRS data available, and neonates with severe congenital malformations.				Study found statistically significant lower CrSO_2_ and higher cFTOE values with no differences in SpO_2_ and HR between favourable outcomes neonatal group and adverse outcome group.	
	GA: 24.8 (adverse outcome group), 30.6 (favourable outcome group)					
Wolfsberger et al.*^50^	Post-hoc analysis of the COSGOD III trial to determine reference ranges for CrSO_2_ in 207 preterm infants with a favourable outcome (survival to discharge without cerebral injury).	INVOS 5100	CrSO_2_, SpO_2_, FiO_2_, HR	< 30 s (n = 118), 30-60 s (n = 58), >60 s (n = 21)	50^th^ centile CrSO_2_ values: 28% (2 m), 35% (3 m), 45% (4 m), 59% (5 m), 66% (6 m), 69% (7 m), 73% (8 m), 75% (9 m), 76% (10 m), 78% (11 m), 80% (12 m), 77% (13 m), 79% (14 m), 78% (15 m)	Post-hoc analysis.
						Heterogeneity of cord-clamping strategies.
	Exclusion: IVH/PVL, congenital malformations or inflammatory morbidities.					Exclusion of inflammatory morbidities.
	GA: 29.7 (23.9-31.9)					
Wolfsberger et al.*^29^	Post-hoc analysis of multiple prospective observational studies exploring the impact of Fetal Inflammatory Response Syndrome (FIRS; via interleukin-6 levels) and cerebral oxygenation in 46 preterm babies.	INVOS 5100 C	cFTOE, CrSO_2_, HR, SaO_2_, IL-6, CRP	30 seconds	cFTOE was significantly lower in the FIRS group compared to the control cohort. There were no significant differences in CrSO_2_.	IL-6 values relatively low despite meeting definition for FIRS.
	Exclusion: congenital malformations					
	GA: 32.1 (FIRS), 32.0 (non-FIRS)					
*Term and Preterm Infants*						
Baik-Schneditz et al. ^72^	Prospective observational study of 122 healthy neonates (14 preterm and 108 term neonates) to measure cerebral oxygen saturations using the MASIMO ROOT machine during the first 15 minutes of birth.	MASIMO ROOT	CrSO_2_, SpO_2_ and HR	Cord clamping approximately 60 s after birth	CrSO_2_ rose from 59.0% (2 m) to 77% (10 m). Cerebral oxygenation reached a stable plateau at minute 7. CrSO_2_ root and cTOI NIRO followed similar trajectories over the 15 minutes, however INVOS measurements tended to show highest values after 5 minutes.	Only near term or term babies born via C-section where included.
	Exclusion: neonates receiving respiratory support or oxygen therapy during immediate postnatal transition and neonates with malformations.					
	GA; 38.7 (38.0-39.0)					
Hendrikx et al.*^52^	Post-hoc analysis of 48 preterm and term neonates reviewing correlations between heart rate, arterial oxygen saturations with rScO_2_ and cFTOE.	INVOS	HR, SpO_2_, ECG, rScO_2_, cFTOE	>30 seconds delayed	2 clusters were identified: Cluster 1 consisted of mainly term neonates whereas Cluster 2 consisted of mainly preterm neonates with lower birth weights.	
	Exclusion: Infants with severe cardiocirculatory, pulmonary or cerebral congenital malformation were excluded.					
	GA median (25^th^ centile, 75^th^ centile): 38.7 weeks (37 weeks, 39.1 weeks).				High coupling was observed between SpO_2_ and rScO_2_. Cluster 1 primarily showed non-linear coupling (silhouette score 0.48) whilst cluster 2 primarily showed stronger linear coupling (0.47).	
					SpO_2_ and cFTOE showed high linear coupling. Coupling was stronger in cluster 2 compared to cluster 1.	
					HR and cFTOE showed high non liner coupling, No difference between clusters.	
Mattersberger et al.*^28^	Post-hoc analysis of 75 neonates to investigate association between cerebral oxygenation and blood glucose.	INVOS 5100	CrSO_2_, cFTOE and blood glucose measured during first 15 minutes of life.	30-60 seconds	CrSO_2_ at 15 minutes for preterm and term babies was 80 ± 12.1 and 83 ± 7.7.	Small sample size for preterm infants.
	Exclusion: congenital malformations, SVD/instrumental deliveries				cFTOE at 15 minutes for preterm babies 0.15 ± 0.10 and term babies 0.14 ± 0.08.	
	GA: 32.8 ± 3.3 (preterm), 38.7 ± 0.8 (term)					
					Statistically significant negative correlation for CrSO_2_ and positive correlation for cFTOE with blood glucose concentration.	
Mattersberger et al. 2023*^27^	Post hoc analysis to investigate potential associations between acid-base and metabolic parameters and CrSO_2_ and cFTOE in 157 neonates (42 preterm and 115 term neonates).	INVOS 5100	pH-value, base-excess, bicarbonate, lactate, CrSO_2_, cFTOE, HR, SpO_2_	Not Documented	No significant differences between term and preterm infants in CrSO_2_ and FTOE. CrSO_2_ at 15 minutes for preterm babies 82 ± 16 and 83 ± 12 for term babies. FTOE was 0.13 ± 0.15 for preterm babies and 0.14 ± 0.14 for term babies at 15 minutes.	Confounding factors not controlled for.
	GA: preterm 34 (3.3), terms 38.9 [1.0]					Small sample size for preterm infants.
	Exclusion criteria: Those not born via Csection, neonates with congenital malformations.				In preterms CrSO_2_, pH and BE had a significant positive correlation, while CrSO_2_ and lactate showed a negative correlation.	
					There was a significant correlation between cFTOE and lactate, while cFTOE, pH and BE showed a negative correlation. In term neonates, there was only a significant positive correlation between FTOE and HCO_3_.	
Pichler et al. ^66^	Prospective observational study of amplitude-integrated EEG and cerebral oxygenation during transition in 63 infants born at >34 weeks after Caesarean section.	INVOS	SpO_2_, rSO_2_, HR, aEEG Vmax measured within the first 10 minutes of life.	Immediate	Endpoint (10 minute) values for rSO_2_ were significantly higher than values for minutes 3-6 in the uncompromised group and minutes 3-8 in the respiratory support group. Overall rSO_2_ values for both groups were lower compared to endpoint between 4-8 minutes of life. Minimum (Vmin) and maximum (Vmax) EEG amplitude values increased with increasing rSO_2_ at 4 minutes of life.	Only infants born via CS included. No other confounders accounted for. No information of missing data and quality control measurements.
	Exclusion: preterm, SVD, congenital anomalies					
	GA: 39 ± 1.4 (uncompromised), 38 ± 1.2 (respiratory support)					
Pichler et al. ^17^	Prospective observational study to determine reference ranges for Regional Cerebral Tissue Oxygen Saturation and Fractional Oxygen Extraction in 381 neonates during immediate transition after birth.	INVOS	CrSO_2_, cFTOE during the first 15 minutes	Not documented	Changes in CrSO_2_ and cFTOE with defining reference ranges and percentile charts in a large cohort of newborn infants without the need of medical support during the first 15 minutes after birth were reported.	Other NIRS devices not assessed.
					In all neonates, median (10th-90th percentiles) CrSO_2_ was 41% (23-64) at 2 minutes, 68% (45-85) at 5 minutes, 79% (65-90) at 10 minutes, and 77% (63-89) at 15 minutes of age. In all neonates, median (10th-90th percentiles) cFTOE was 33% (11-70) at 2 minutes, 21% (6-45) at 5 minutes, 15% (5-31) at 10 minutes, and 18% (7-34) at 15 minutes of age.	
	Exclusion: congenital malformations, respiratory support, instrumental delivery.					
	GA: 40 ± 1.3 (NVD, 39.0 ± 0.9 (CS), 34.9 ± 1.4 (preterm CS)					
Suppan et al. ^53^	A prospective observational study to investigate impact of HbFc on rScO_2_, cFTOE and SpO_2_ in 109 neonates during the first 15 min after birth.	Not Documented	CrSO_2_, cFTOE, SpO_2_, HR, Hb and HbFc during the first 15 minutes	Not documented	CrSO_2_ and SpO_2_ values in preterm neonates were higher compared to term infants until the 5th minute after birth and the calculated cFTOE values were lower until minute 4.	Small preterm sample size
						Only studied neonates born via CS.
	Exclusion: NVD/instrumental deliveries, congenital malformations, missing blood analysis/insufficient NIRS data.				Positive correlations of both HbFc and Hb with CrSO_2_ and negative correlations of HbFc and Hb with cFTOE in the first minutes after birth in preterms. In contrast, there were no significant correlations between the same parameters in term neonates.	
	GA preterm: 34.4					
	GA term: 39.0					
Wolfsberger et al.*^25^	Post-hoc analysis of prospective observational studies evaluating the effect of pCO_2_ on CrSO_2_, cFTOE, TOE, HR in 84 term and 11 preterm infants born via Caesarean section.	INVOS 5100 C	CrSO_2_, cFTOE, cTOE, pCO_2_, pO_2_, HR, BP, blood glucose, SpO_2,_	Not documented	High pCO_2_ values were associated with lower CrSO_2_ and higher cFTOE/cFOE values in preterm babies, with no correlations found in term babies.	Uneven sample sizes. Cerebral blood flow/volume not examined.
	Exclusion: congenital malformations, need for respiratory support or supplementary oxygen.					
	GA: 34.8 weeks (preterm), 39.0 (term)					
*Umbilical Cord Management Strategies*						
Katheria et al. ^64^	Randomised cluster cross-over trial comparing intact umbilical cord milking to early cord clamping, evaluating changes in StO_2_, SpO_2_, FiO_2_, HR. Analysis of 34 patients recruited for MINVI trial.	FORE-SIGHT	StO_2_, SpO_2_, HR, FiO_2_ measured for first 10 minutes of life. NIRS blinded to practitioners.	Early clamping <60 s or cord milking (4 times)	No significant differences in NIRS outcomes. Non-vigorous infants receiving umbilical cord milking received lower amounts of supplemental oxygen compared to early cord clamping.	Small subgroup of infants in level III NICUs. Not blinded to intervention (cord management).
	Exclusion: <35 weeks, vigorous at birth					
	GA: 38.1 ± 1.5 (cord milking), 38.95 ± 1.7 (early clamping)					
Kuehne et al. ^63^	Randomised control trial comparing the effect of extrauterine placental perfusion or delayed cord clamping on haematocrit and cerebral oxygenation in 60 infants born via elective Caesarean section	FORESIGHT	HR, SpO_2_, CrSO_2_, FiO2, mean airway pressure, mean haematocrit level	0 seconds (DCC group).	At 5 minutes of life, infants receiving EPP had statistically higher SpO2 (15.3%) and CrSO_2_ levels (11.3%) in comparison to the DCC group.	Small sample size; may not be reflective of safety of EPP.
				EPP group – until onset of stable respiratory pattern and HR >100bpm	Mean haematocrit levels were similar for both groups.	Study resuscitation protocol (use of EPP or incremental increase in CPAP pressure) is unlikely to be routinely used in other centres.
	Exclusion: <23 + 6 weeks, birthweight >1500 grams, SVD/instrumental delivery/emergency CS, fetal or maternal risk (compromise/general anaesthesia), congenital malformations, placental pathology (increta/accreta/abruption/praevia with haemorrhage) and monochorionic multiples					
					Secondary neonatal outcome parameters (death/intubation/BPD/NEC/SIP/PVL/IVH/ROP were similar for both groups.	
	GA: 27 + 6					
					EPP CrSO_2_: 52.9 (1 m), 53.2 (2 m), 54.6% (3 m), 62% (4 m), 65.3% (5 m), 74.4% (6 m), 78.5% (7 m), 76.3% (8 m), 81.4% (9 m), 81.2% (10 m)	
					DCC CrSO_2_: 37% (1 m), 49.8% (2 m), 49.4% (3 m), 50.3% (4 m), 51.9% (5 m), 56.5% (6 m), 62.8% (7 m), 65.1% (8 m), 71.3% (9 m), 74.6% (10 m)	
Pichler et al.*^61^	Prospective Observational study to investigate impact of DCC on cerebral oxygenation of 76 preterm neonates born via CS section.	INVOS 5100 C	HR, SpO2, CrSO_2_, cFTOE	DCC was defined as 60 seconds and early cord clamping was defined as <30 seconds.	Mean cFTOE was significantly lower in early cord clamping between minutes 3-6 of life. Delayed cord clamping CrSO_2_ values were significantly lower at minute 5 of life.	Small sample size.
	DCC and ECC groups were matched for GA, birth weight and sex.					
	Exclusion: SVD, congenital malformations, antepartum haemorrhage.					
	GA: 32 (31-33) in DCC group, 32 (31-33) in ECC group					
Schwaberger et al. ^62^	Randomised control trial evaluating cerebral tissue oxygenation index between 71 vaginally delivered term neonates with deferred/physiological-based cord clamping (PBCC) or a standard time-based cord clamping <1 minute.	NIRO 200 NX DP	Cerebral tissue oxygenation index (cTOI), change in cerebral blood volume (CBV), SpO_2_ and HR within first 15 min after birth.	Physiological:	There were no significant group differences in the primary outcome parameter cTOI or in the secondary outcome parameters ΔCBV, SpO_2_ and HR during the first 15 mins after birth	Only studied term neonates and neonates born vaginally.
				After regular respiratory rate >30 (estimated 2-4 minutes)		Investigator bias.
	Exclusion criteria: maternal peripartum complications, congenital malformations and need for respiratory support within 15 min after birth resulting in immediate cord clamping.				Time of cord clamping in PBCC group is 275 (197-345) sec and then 58 (35-86) seconds.	
				Control: 30-40 s		
	GA: 39 in PBCC and 40.0 in control group					
Satar et al. ^65^	Prospective, cross-sectional, randomised study of 84 term infants to evaluate the effect of 60 second or 180 second delayed cord clamping on cerebral oxygenation.	INVOS 5100 C	CrSO_2_ at 5 min and 10 min, HR, Peripheral oxygen saturation, APGAR, HR	2 groups: 60 seconds delayed cord clamping and 180 second delay in cord clamping.	There is no significant difference in CrSO_2_ values at 5 min and 10 min between 60 s and 180 s delayed cord clamping groups (p = 0.86 at 5 min and p = 0.23 at 10 min).	No gestational age given.
	Exclusion: Additional problems in perinatal follow-up, placental abnormalities (placenta previa/accreta), need for resuscitation after birth, family refusal to participate in the study, preterm birth, SGA, LGA babies, multiple pregnancies and congenital malformations				There was a significant difference in the proportion of neonates with targeted CrSO_2_ values at 5 minutes: 59.5% of infants in the 60-second DCC group had abnormal CrSO_2_ values, compared to just 19% in the 180-second DCC group (p < 0.001).	
					Target crSO2 measures were accepted as 60-80%.	
	GA: 37-42 weeks					
*Interventions in the Delivery Room*						
Pfurtscheller et al.*^59^	Post-hoc analysis of four observational studies examining correlations between cardiac output with cerebral oxygenation and cerebral fractional tissue extraction. Total of 286 babies including 79 preterm babies.	INVOS 5100 C	SpO2, CrSO_2_, cFTOE, SABP were measured for the first 15 minutes of life.	30-60 seconds	Mean CrSO_2_ in preterms not requiring respiratory support was 86% (81-93) between minutes 13-15 of life, which was significantly higher (p < 0.001) than preterms with respiratory support (76% [69-76]).	No blood gas parameters were recorded.
	Exclusion: low umbilical artery pH ( < 7.0), congenital malformations, SVD				cFTOE was also lower in preterms without respiratory support (0.1 [0.03-0.13]) than preterms with respiratory support (0.16 [0.16-0.23]), which was also significant (p = 0.004)	
	GA: 29.4 ± 3.7 (preterm respiratory support), 34.9 ± 1.3 (preterm without support), 38.7 ± 0.7 (term with respiratory support), 38.7 ± 0.8 (term without respiratory support)				Significant positive correlation between CO and CrSO_2_ and then cFTOE, in preterms requiring respiratory support (p < 0.05).	
Pichler et al. ^44^	Randomised controlled pilot study to assess effectiveness of cerebral regional tissue oxygen saturation to guide respiratory and supplemental oxygen support to reduce cerebral hypoxia in 60 preterm neonates during resuscitations after birth.	INVOS	CrSO_2,_ SpO_2_, HR and cFTOE started after birth and until 15 minutes after birth.	30-60 seconds	Presents CrSO_2_ between 2-15 minutes between study and control group.	Missing data of CrSO_2_ resulting in including only 52% of neonates in primary analysis
					In the NIRS-visible group burden of cerebral hypoxia in % minutes, CrSO_2_ was halved, and the relative reduction was 55.4%. Cerebral hyperoxia was observed in NIRS-visible group in 3 neonates with supplemental oxygen and in NIRS-not-visible group in 2	
	Exclusion criteria: Congenital malformations or palliative pathway					
	GA: 29.8 ± 3					
Pichler et al. ^45^	Randomised control trial following after Pichler et al. ^44^ investigating whether monitoring of cerebral tissue oxygen saturation using NIRS in addition to routine monitoring during immediate transition of 607 preterm neonates would improve survival without cerebral injury.	INVOS 5100	CrSO_2_, SpO_2_, FiO_2_, HR	Not documented	CrSO_2_ levels were comparable in both groups. Monitoring cerebral tissue oxygen saturation along with targeted interventions in preterm neonates ( < 32 weeks gestation) during immediate transition and resuscitation after birth did not lead to significantly improved survival rates without cerebral injury compared to standard care alone.	Most babies born via CS. Blood gas parameters not included.
	Exclusion criteria: congenital malformations or prenatal cerebral injury					
	GA: 28.9 (NIRS group), 28.6 (control).					
Pocivalnik et al. ^60^	Prospective observational study investigating impact of oropharyngeal suctioning immediately after delivery on cerebral tissue oxygenation in 72 term neonates after elective CS.	INVOS	rSO_2_brain, pre/post ductal peripheral muscle (rSO_2_pre/rSO_2_post) tissue oxygenation, HR, SpO_2_ during the first 15 minutes of life	Not documented	Suctioning had no effect on CrSO_2_ during the first 15 minutes of life.	Small sample size
	Exclusion: Congenital malformations, need for respiratory support, normal vaginal deliveries				Cerebral regional oxygen saturation at 2 mins was 44( ± 21) and 41 ( ± 13), at 7 mins was 67(±16) and 67( ± 16) and at 10 mins was 75( ± 14) and 78( ± 11) in the control group and suction group respectively.	
	GA: 38.9 (control) and 39.1 (suction group).					
Schwaberger et al. ^54^	Randomised control trial investigating effect of sustained lung inflations during neonatal resuscitation in 40 preterm infants.	NIRO-200-NX	CBV, cTOI during immediate postnatal transition.	Immediate	cTOI was significantly increasing within the study period without any differences between both groups.	Only infants born via CS included.
						Exclusion of ELBW.
	Exclusion criteria: infants not born via CS, extremely low birth weight infants				CBV behaviour was significantly different in infants with SLI compared to control group. CBV significantly decrease from minutes 3 to 15 in control group whilst CBV was unchanged in SLI group.	No continuous monitoring of pCO_2_
	GA: 32 + 1 ( ± 2)					
Schwaberger et al.*^58^	Post-hoc analysis of four studies evaluating changes in cerebral blood volume and the need for respiratory support at birth in 204 infants (45 preterm/159 term).	NIROS 200-NX	Change in CBV, cTOI, SpO_2_, HR	30 seconds	Statistically significant decrease in CBV within 15 minutes after birth. This was smaller in babies requiring respiratory support than babies without support, which was significant at minutes 2, 6 and 7.	Only the factors respiratory support and Gestational Age were considered in finding best model.
	Exclusion criteria: NVD, congenital malformations, inherited disorders of metabolism, palliative care pathways, use of sustained lung inflations.				Lower cTOI levels were observed in infants with respiratory support in comparison to those without respiratory support. (p = 0.023), but with no significant differences between gestation.	Groups had significant differences in GA and hence the influence of GA could not be sufficiently investigated.
						Only neonates born by CS were included.
	GA: 33.5 ± 2.1 in preterm infants, 38.9 ± 0.9 in term infants.					

*BP* blood pressure, *BPD* bronchopulmonary dysplasia, *CS* Caesarean section, *CGA* corrected gestational age, *CrSO2/rSO2/rcO2* cerebral regional oxygen saturations, *CtSO2/StO2* cerebral tissue oxygen saturations, *cTOI* cerebral tissue oxygenation index, *cFTOE* cerebral fractional tissue oxygen extraction, *CBV* cerebral blood flow, *CrUSS* cranial ultrasound scan, *EEG* electroencephalogram, *ELBW/VLBW* extremely low/very low birthweight, *FiO2* fraction of inspired oxygen, *GA* gestational age, *Hb* haemoglobin, *HbFcHHb* deoxygenated haemoglobin, concentration of fetal haemoglobin, *HR* heart rate, *IQR* interquartile range, *IUGR* intrauterine growth restriction, *IVH* intraventricular haemorrhage, *MAP/MABP* mean arterial blood pressure, *NEC* necrotising enterocolitis, *NICU* neonatal intensive care unit, *NIRS* near-infrared spectroscopy, *SVD/VD* spontaneous vaginal delivery/vaginal delivery, *SpO2* peripheral oxygen saturations, *SLI* sustained lung inflations, *THI* tissue haemoglobin index. Studies marked with an asterisk (*) were post-hoc analyses and were not included in the final statistical analysis.

Most studies observed healthy term babies: only 1282 (31.1%) patients from 12 studies were premature. Babies needing respiratory support or resuscitation were excluded in 16 studies, whilst 9 studies evaluated interventions including cord clamping (*n* = 3), respiratory support or supplemental oxygen (*n* = 2), oropharyngeal suctioning (*n* = 1), sustained lung inflations (*n* = 1) and interventions to achieve target cerebral saturations (*n* = 2).

Cerebral oxygen saturation values were measured in 34 studies and FTOE measured in 12 studies whilst 6 studies explored cerebral tissue oxygenation index (cTOI). 18 studies used INVOS, 7 used FORE-SIGHT, 7 used NIRO and six studies used other devices. Three studies included FTOE in preterm babies. Key confounder data such as blood gas parameters or cerebral hemodynamic measurements were rarely included although NIRS protocols were well described (Fig. 2).

**Fig. 3:**
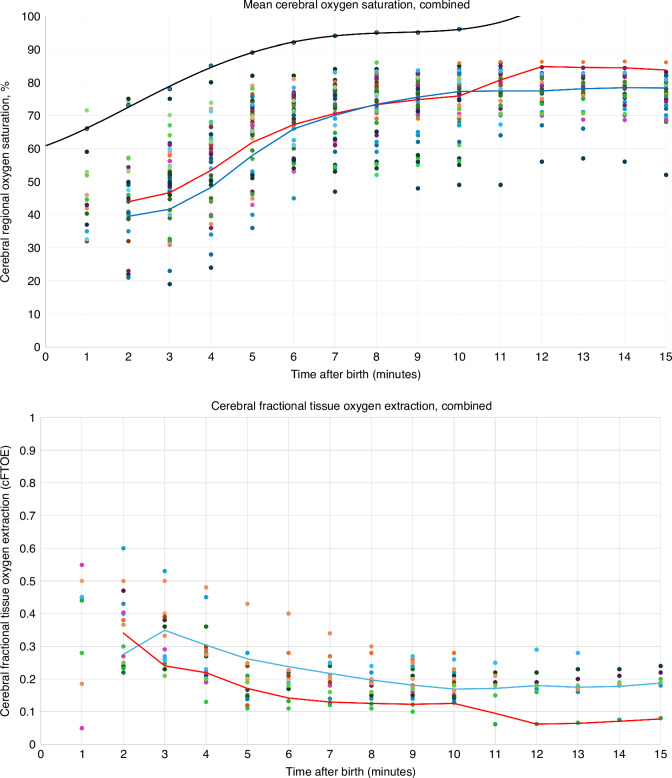
Mean Cerebral Oxygenation and Cerebral Fractional Tissue Oxygen Extraction values for term (red) and preterm (blue) babies compared to combined reference ranges for peripheral oxygen saturation values^13^(black).

Only one study measured cFTOE exclusively in preterm babies,^12^ reporting an extremely low cFTOE value at 1 minute of life (0.05) which was excluded from statistical analysis. Pooled data from the reviewed studies (*n* = 43); with 4111 babies (Fig. 3) demonstrate trends in mean CrSO_2_ and cFTOE for both term and preterm babies. This was compared to reference ranges for peripheral arterial saturations (SpO_2_) published by Dawson et al.^13^ Cerebral oxygen saturations were on average 22.5% lower than peripheral saturations in preterm babies and 20.6% lower in term babies in the first ten minutes after birth. In preterm babies CrSO_2_ reached a steady state of 70–77% by 10 to 15 minutes after birth and 72–85% in term babies respectively. ANOVA testing found no significant differences between preterm and term infants in CrSO_2_ (*p* = 0.54) and cFTOE (*p* = 0.50). cTOI was not analysed quantitatively due to scarcity of data.

Seven prospective studies^14–20^ with aggregate 886 term and 40 preterm infants published reference ranges. Sex differences were explored in one study, with no significant differences in cTOI although male neonates had a higher cardiac output.^21^

Multiple studies explored physiological parameters during the neonatal transition. Relationships between cerebral oxygen and cardiovascular/cerebrovascular haemodynamics were explored by Noori et al.^22^ observing that as CrSO_2_ rose, there were reductions in middle cerebral artery flow and heart rate coupled with increased shunting across the ductus arteriosus and improved stroke volume.^22^ Faucheré et al.^23^ also observed simultaneous reductions in cerebral deoxygenated haemoglobin whilst TOI peaked at a steady state by around 8 min of life. Baik-Schneditz et al. evaluated the relationship between cardiac output and cTOI, finding no statistically significant correlation between the two measures.^24^

Elsewhere, the effects of carbon dioxide on cerebral oxygenation were reviewed in a post-hoc analysis of 95 term and late preterm babies by Wolfsberger et al.: in preterm babies, higher pCO2 values were negatively correlated with CrSO_2_, whilst a positive correlation was observed between CrSO_2_ and cFTOE.^25^ One post-hoc analysis evaluated the impact of prenatal tobacco exposure on cerebral oxygenation, finding that CrSO_2_ was lower and cFTOE was higher in babies exposed to prenatal until minute 5 after birth.^26^

Mattersberger et al.^27^ explored relationships between CrSO_2_ and cFTOE with metabolic parameters in a post-hoc study, finding that in comparison to term babies, pH, base excess and lactate were all significantly lower in preterm babies. In preterm babies, CrSO_2_ and lactate had a negative correlation whilst cFTOE and lactate had a positive correlation. Meanwhile there was only a positive correlation between cFTOE and bicarbonate in term babies. The effect of blood glucose was also explored by Mattersberger et al.^28^ in a post-hoc secondary outcomes study, concluding that there was a significant negative correlation between blood glucose and CrSO_2_ (with a significant positive correlation between blood glucose and cFTOE), which was more pronounced in preterm babies. This was thought to be because of vasodilatation secondary to low blood glucose and its subsequent effects on cerebral blood flow.

Lastly, Wolfsberger et al. conducted a secondary outcome analysis of 46 preterm babies exploring the correlation between Fetal Inflammatory Response Syndrome (FIRS; defined as interleukin 6 levels > 11 pg/mL) and cerebral oxygenation, demonstrating no significant differences in CrSO_2_ values but lower cFTOE values at minutes 2 to 4 after birth in the FIRS group compared to the non-FIRs group.^29^

### NIRS and term Infants

Term infants were the most studied group, accounting for 31 studies and a total of 2442 babies. Many of these trials were formative observational trials using predefined cohorts of healthy babies born via uncomplicated pregnancies. As a result, these trials have limited applicability in clinical practice as this is not representative of the patient cohort in most neonatal units.

The largest trial in term babies alone was by Xue et al. which examined the effects of maternal anaesthetic factors on cerebral oxygenation in 418 babies born via elective Caesarean section, demonstrating a rise from around 49% at 2 minutes to a steady state between 55.7 and 81.0% at 7 to 8 min of life.^30^ Meanwhile, the relationship between cerebral activity (measured via amplitude-integrated EEG) and cerebral oxygenation was explored in one study of 59 babies born via elective Caesarean section, finding that babies with lower cerebral activity had significantly lower CrSO_2_ values until minute 11, with higher cFTOE values until minute 10^31^.

Ergon et al.^32^ explored the effects of body position on cerebral oxygenation in 60 healthy term infants, finding no significant differences in CrSO_2_ or cFTOE if babies were in the supine, prone, left or right lateral positions.

Reference ranges have been published in several studies using various NIRS devices,^14–16^ demonstrating the establishment of a steady state by 10 minutes of life in CrSO_2_ (60–76%), cTOI (68-69%) and cFTOE (0.22). Similarly, in a study of 63 term babies, Kratky et al. also reported increases in CrSO_2_ from 39% to 75% and a decrease in cFTOE from 0.47 to 0.22 between 2 to 15 minutes after birth.^33^

Baik et al.^19^ demonstrated that a steady state was achieved by 10 minutes of life for both cTOI (61–83%) and cFTOE (0.13–0.37) in 140 babies. Meanwhile, Nastase et al. described an increase in CrSO_2_ from 35.5% to 71.1% and a decrease in cFTOE from 0.42 to 0.19 between 1 to 10 minutes of life^16^ in 74 babies. Urlesberger et al. also reported a similar increase in CrSO_2_ from 44% to 76% between 3 and 7 min after birth in a study of 59 babies.^34^ Using the MASIMO ROOT device, Carnicero et al. studied 44 healthy newborns, finding that although cerebral oxygenation values also followed the same trajectory, values using the MASIMO ROOT were higher compared to NIRO measurements.^20^

Meanwhile, using the KN-15 device, Ozawa et al.^18^ reported that median CrSO_2_ increased from 44.6% at 1 min and stabilized to 56.2% by 10 min of life in 127 babies born via elective Caesarean. Similarly, Kato et al.^15^ investigated trends in CrSO_2_ of 100 babies born via vaginal delivery or Caesarean section, demonstrating that median CrSO_2_ levels rose from 43% at 1 minute to 57% at 10 min. Using the same device, Watanabe et al. also described significant rises in CrSO_2_ from 43% to 49% between minutes 1 to 4 of life.^35^

Lastly, Schwaberger et al. measured cerebral oxygen saturations alongside cerebral blood volume in an observational study involving 109 babies born via elective Caesarean section, demonstrating a decrease in cerebral blood flow in the first 15 minutes of life, alongside a rise in cTOI to a steady state of approximately 73%.^36^

### NIRS and modes of delivery

Several studies have used NIRS to evaluate the differences in cerebral oxygenation measures between modes of delivery.^37–41^ Healthy term infants born via caesarean section (CS) were the most studied patient group. Isobe et al.^40^ examined cerebral haemoglobin oxygen saturation (SbO_2_) using IMUC-7000, in 20 healthy term infants delivered vaginally and 6 infants born via elective CS. They observed similar initial increases in SbO_2_ from 2 mins to 8.5 minutes after birth in both groups. However, beyond this time, SbO_2_ significantly dropped in the Caesarean group to 57.5% at 15 minutes, while the vaginal delivery group steadily rose to 66% at the same time (*p* < 0.05).

Similarly, Farag et al.^14^ found that CS was the most significant factor affecting cerebral oxygenation during transition period. Babies born via CS had significantly higher CrSO_2_ and lower FTOE at 5 minutes and 10 min after birth than those born vaginally. Additionally, the rate of CrSO_2_ rise from 1 to 5 min of life was significantly higher among the vaginal group (36%) than the caesarean group (22%) with *p* < 0.001. Morimoto et al.^41^ found that cerebral blood volume (CBV) was significantly higher in VD infant born group than in CS during the first 4 minutes of life. Despite this variation, they found no significant differences in cerebral hemoglobin oxygen saturation between the groups, suggesting that oxygenation mechanisms remain robust across different delivery modes.

Meanwhile, Karen et al.^39^ compared the influence of vacuum-assisted deliveries and CS on cerebral oxygenation in 15 neonates, compared to results of 19 infants born via c-section from Fauchere et al.^23^ They found that cerebral tissue oxygenation index (cTOI) values were 11.6 ± 6.3% lower in CS (53 ± 3%) than vacuum-assisted deliveries (65 ± 3%) in the first 0–5 min, which was statistically significant (*p* = 0.3). No statistically significant differences were found between the groups after 5 min of life (*p* > 0.1). Newborns delivered by vacuum extraction had significantly higher tissue haemoglobin index (THI) 10 to 15 min after birth.

In contrast, Urlesberger et al.^38^ found no statistically significant differences in CrSO_2_ or FTOE in respect to mode of delivery, involving 63 term infants after spontaneous delivery and 44 infants after Caesarean section. Likewise, when using the FORE-SIGHT device, Almaazmi et al.^37^ found no difference in the changes cStO_2_ from 2^nd^ minute after birth of healthy term infants born via spontaneous birth, CS and assisted delivery.

A post-hoc analysis of 62 babies^42^ explored the effects of maternal general or spinal anesthesia on cerebral oxygenation, with no significant differences found in CrSO_2_ or cFTOE values in both preterm and term neonates, despite the general anesthesia group having higher FiO_2_ requirements between minutes 3–9 after birth.

### NIRS in premature neonates

Twelve studies included 1282 preterm infants in their analysis. Five studies^43–47^ exclusively included babies born < 32 weeks. Prematurity and the need for respiratory support or resuscitation were common exclusion criteria in the published literature. Baik et al.^48^ included 186 preterm infants (of a total 462) in a large cohort study, concluding that late preterm infants (mean GA 31.0 weeks) had significantly lower MABP and FTOE values in comparison to the term infants, suggesting impairments to cerebral autoregulation in the preterm brain. Fuchs et al.^12^ published reference ranges for 51 very low birthweight infants ( < 1500 grams; GA 27.8 weeks), demonstrating an increase in CrSO_2_ from 37% at 1 minute after birth to a steady state between 61 to 84% approximately 7 min after birth.

Ertekin et al.^49^ evaluated differences in cerebral autoregulation in a study of 39 babies (mean CGA 34.7 weeks) finding no significant differences in CrSO_2_ or cFTOE between healthy term and late preterm infants requiring no respiratory support at birth.

Pichler et al.^17^ published reference ranges in cohort study of healthy newborns, including 27 late preterm infants (GA 34.9 weeks). Whilst preterm infants had significantly lower heart rates than their term counterparts, there were no significant differences in CrSO_2_ or FTOE values. Pichler et al.^44^ also explored trends in cerebral oxygenation in late preterm infants with (GA 29.8 weeks) and without visible NIRS data (GA 29.2 weeks) guiding oxygen delivery. There were no significant differences in rates of mortality or morbidity. This was followed by the COSGOD III trial,^45^ the largest trial to date investigating cerebral oxygen saturations in the delivery room. In this trial, 607 preterm infants < 32 weeks gestation were randomised to either a NIRS-visible or control group. Cerebral oxygenation values were used as an indicator to increase FiO_2_ or escalate respiratory support. There were no statistically significant differences in survival without cerebral injury. There were no significant differences in the need for resuscitative measures such as intubation, chest compressions, surfactant, intravenous fluids or adrenaline.

Equally, there were also no significant differences in the incidence of secondary outcomes including respiratory distress syndrome, necrotising enterocolitis, culture-proven sepsis, bronchopulmonary dysplasia, retinopathy of prematurity or persistent ductus arteriosus.

A secondary outcome analysis of the COSGOD III trial^50^ published centile charts for 207 infants with a favourable outcome (excluding cerebral injury, death or inflammatory morbidities). Over 90% of this cohort required respiratory support, with lower SpO2 and CrSO_2_ values being reported when compared to the existing literature base.^13,17^ This is likely to be a better representation of extreme preterm physiology when compared to stable cohorts of moderate-to-late preterms used to establish reference ranges.

A further retrospective comparative analysis by Wolfsberger et al.^51^ showed that preterms (gestational age ≤ 32 weeks and birth weight ≤ 1500 g) with adverse outcomes (BSID-III < 70 or mortality) had significantly lower GA, lower CrSO_2_ and higher cFTOE in the transition period without significant changes in SpO_2_ and HR.

A post-hoc analysis by Hendrix et al.^52^ demonstrated a differential coupling effect between heart rate, arterial oxygen saturations with cerebral oxygenation. They identified two clusters: Cluster 1 (mostly term neonates) and Cluster 2 (mainly pre-term neonates). The study revealed that coupling of SpO_2_ and HR with cerebral oxygenation was stronger and more linear in neonates with lower GA, suggesting that gestational age significantly influences how these parameters interact in the immediate postnatal transition. Suppan et al.^53^ evaluated fetal haemoglobin concentration (HbFc) and cFTOE in 109 babies, of whom 19 were late preterms (GA 34.4 weeks). HbFc values were significantly higher in preterm infants, suggesting better oxygen-carrying capacity, with higher CrSO_2_ values and lower cFTOE values, which may be due to impaired autoregulation or lower preterm oxygen consumption during the neonatal transition period.

Further studies^43,54^ have investigated CrSO_2_ levels in preterm infants during delivery requiring different degrees of oxygen; and between standard and sustained lung inflations, albeit without a term comparator. Preterm infants receiving lower concentrations of oxygen had higher CrSO_2_; there was no difference in CrSO_2_ between standard care and sustained inflations performed at birth. Mattersberger et al.^27^ conducted a post-hoc analysis evaluating metabolic parameters, finding no differences in cerebral oxygenation between 115 term and 42 late preterm infants (GA 34.0 weeks). In another secondary outcome study examining blood glucose, Mattersberger et al.^28^ found no differences in CrSO_2_ or cFTOE between 50 term and 25 late preterm infants (GA 32.8 weeks). Similarly, a post-hoc study by Wolfsberger et al.^25^ exploring the effects of carbon dioxide levels found no significant differences between 84 term infants and 11 late preterm infants (GA 34.8 weeks).

In a further post-hoc analysis, Beik-Schneditz et al.^55^ compared 45 IUGR neonates (GA 33.1 weeks) with and 135 sex-matched AGA neonates (GA 33.3 weeks) born via Caesarean section. The study found no significant difference in CrSO_2_ between the groups although cFTOE was significantly lower in IUGR neonates at 5–9 minutes and 11–13 minutes after birth.

In a retrospective analysis of four studies, Breseti et al.^56^ investigated the relationship between bradycardia, hypoxaemia and cerebral oxygenation in 150 preterm infants (mean GA 33 weeks). The results demonstrated that within the first 15 minutes of life, bradycardia alone only had a significant effect on peripheral oxygen saturations (SpO_2_), whilst the effects of bradycardia in conjunction with hypoxaemia led to significantly lower CrSO_2_ and cFTOE values.^56^

### NIRS and interventions in the delivery room

The effects of respiratory support on cerebral oxygenation during the neonatal transition in delivery room have been investigated in several trials. Binder et al.^57^ compared the effects of non-invasive respiratory support to normal transition in 42 infants aged 30–37 weeks delivered by Caesarean section, demonstrating significantly lower levels of cerebral oxygenation and cFTOE in the respiratory support group. In a post-hoc analysis by Schwaberger et al.^58^ neonates born via elective Caesarean section receiving respiratory support had statistically lower cTOI values and smaller changes in CBV than neonates without respiratory support. Moreover, in another post-hoc analysis by Pfurtscheller et al.^59^ cardiac output was positively significantly correlated with CrSO_2_ and negatively with cFTOE in 59 preterms with respiratory support, further supporting theories of impaired cerebral autoregulation. No significant associations were found between preterms without respiratory support and term babies.

Associations with different oxygen requirements have also been investigated: Kenosi et al.^43^ studied 47 preterm infants born < 32 weeks and demonstrated that infants receiving lower fraction of inspired oxygen concentrations (FiO_2_) had significantly higher cerebral oxygen saturation levels. Pichler et al.^44^ used CrSO_2_ to guide respiratory support during the newborn transition, which was later fully realised in the COSGOD III trial,^45^ a multi-centre study of 607 neonates that demonstrated no statistical differences in survival or CrSO_2_ following the introduction of a dedicated NIRS-based treatment guideline in the delivery room.

Pocivalnik et al.^60^ explored the effects of oropharyngeal suctioning on CrSO_2_ immediately after delivery with no significant differences identified. The use of sustained lung inflations (SLI) lasting up to 15 s was investigated by Schwaberger et al.^54^ in a randomised controlled trial of 40 preterm infants (mean gestation 32 + 1 weeks), finding that cerebral blood volume was unchanged in the SLI group in comparison to a significant decrease in the control group. There were no differences in cerebral tissue oxygenation, peripheral oxygen saturation, blood gas parameters, mortality or morbidity.^54^

### NIRS and Cord management

Reports of cord clamping practices were limited: the duration was not recorded in 22 studies, whilst the cord was clamped immediately in 5 studies and between 30-60 seconds in the remaining studies (Table 1).

Pichler et al.^61^ found that in a cohort of 38 preterms born via CS, delayed cord clamping (at 60 seconds) was associated with higher cTOI from min 3 to min 6 and lower CrSO_2_ in 5 min, in comparison to 38 GA, birthweight matched preterms with early cord clamping ( < 30 seconds). Schwaberger et al.^62^ compared a physiological approach of deferred cord clamping after stable regular breathing with a standard time-based cord clamping control group ( < 1 min of life). There were no significant differences observed in either cTOI or CBV during this period (p = 0.319, p = 0.814 respectively).

Kuehne et al.^63^ compared cerebral oxygenation saturations between those neonates with extrauterine placental perfusion (EPP) approach for physiological based cord clamping very low birth weight (VLBW) to those with delayed cord clamping. In EPP, they clamped umbilical cord when infants showed regular spontaneous breathing, stable heart rate ( > 100 beats per minute) and adequate oxygen saturations. In EPP group, from 1 min to 10 min, Cerebral regional oxygenation was higher in EPP group than in DCC group, with significantly higher readings from 4 min to 10 min. There were no significant differences in other neonatal outcomes including haematocrit levels and other outcomes such as pneumothorax, necrotising enterocolitis and IVH.

Katheria et al.^64^ investigated cerebral haemodynamics in non-vigorous term newborns undergoing umbilical cord milking (UCM) compared to early cord clamping. Their findings indicated that neonates in the UCM group required lower FiO_2_ while maintaining similar cerebral oxygenation levels to those in the early clamping group. Although cerebral oxygen saturations did not differ significantly between the groups, the reduced FiO_2_ requirement in the UCM group suggests that UCM may enhance placental perfusion, potentially improving systemic or pulmonary blood flow.

Satar et al.^65^ found no statistically significant differences in cerebral oxygenation for those with 60 s DCC and 180 s DCCs for values at 5 min and 10 min. However, they found that 59.5% (25 infants) in the 60 s DCC group and 19.5% (8 neonates) in the 180 s DCC group had abnormal CrSO_2_ values outside of the 60-80% target.

### NIRS and the prediction of long-term outcomes

There is a scarcity of data exploring the specific association between long-term outcomes and the neonatal transition, with short-term data reported in only four studies. Baik et al.^47^ explored interventricular haemorrhage formation in a matched study of 24 extreme preterm infants, finding no significant differences in maternal risk factors or indication for delivery. Between 6 and 15 min of life, the mean CrSO_2_ values were significantly lower in the IVH group than in the non-IVH group despite no significant differences in SpO_2_, heart rate or blood pressure. Cardiac output, blood gas parameters and cerebral blood flow, however, were not measured. Additionally, Schwaberger et al.^54^ evaluated the use of sustained inflation breaths at birth, but did not find any differences in mortality, morbidity or cerebral tissue oxygenation following SLI.

Meanwhile, the Neu-Prem trial^46^ used amplitude-integrated electroencephalography (aEEG) in association with cerebral oxygen tissue saturation to predict infants at risk of IVH development in 127 babies born < 32 weeks. Whilst aEEG was not predictive of IVH or death, cerebral tissue oxygenation in babies who developed severe IVH or death was significantly lower in minutes 8 to 10 after birth in comparison to babies with no IVH or low grade IVH. These results were limited by the sample size (*n* = 4) of the severe IVH/death group.

One trial of 42 term and late preterm infants^66^ also explored the relationship between respiratory compromise at birth, cerebral oxygenation and cerebral activity (measured through aEEG). A significant correlation was noted between mean minimum and maximum amplitude values with rSO_2_ values in the uncompromised group at minute 4 after birth, whilst rSO_2_ was lower between minutes 4 to 8 in the respiratory support group when compared to the uncompromised group.

Pichler et al.^44,45^ explored the effect of NIRS-visible data on outcomes such as survival without cerebral injury, bronchopulmonary dysplasia, necrotising enterocolitis, retinopathy of prematurity, spontaneous intestinal perforation, persistent ductus arteriosus and culture-proven sepsis in two studies including a total of 667 babies. There were no significant differences in outcomes in the NIRS-visible and control groups.

One ancillary study to the COSGOD III trial^67^ analysed cerebral oxygenation values for 171 babies with fidgety motor movements as a potential indicator of cerebral palsy. These movements were identified via video recordings between 6 to 20 weeks after birth and classified as either normal or pathological. NIRS monitoring during the neonatal transition had no significant impact on mortality or development of fidgety movements in the study group.

There is some evidence to suggest a link between poor cerebral oxygenation and adverse neurodevelopmental outcomes: a retrospective post-hoc analysis of 42 babies found that babies with adverse outcomes (defined as death or survival with a Bayley Score of Infant Development II/III < 70) had lower CrSO_2_ (in 10 out of the 14 minutes studied) and higher cFTOE levels than babies in the favorable outcome group.^51^

## Discussion

The results from this review demonstrate the normative reference ranges of cerebral oxygenation in the delivery room during immediate neonatal transition; there was no significant differences between trends in cerebral oxygenation between preterm and term babies.

Interest in the use of NIRS to further examine the neonatal transition period is growing: Schlatzer et al. published a recent qualitative review^68^ concluding that cFTOE plays a valuable role in illustrating the complex relationships between haemodynamic, metabolic, respiratory and perinatal factors in the newborn transition.

To the best of our knowledge, this paper is the first to publish aggregated reference ranges across gestations for cerebral oxygenation, the first review of CrSO_2_ during the newborn transition and the largest review of this topic to date.

This offers a unique insight into the real-time processes of cerebral oxygenation during neonatal transition, suggesting comparable physiology between term and late preterm babies. Aggregated mean values were weighted to account for differences in population size.

In preterm babies, CrSO_2_ was on average 22.5% lower than peripheral saturations and in term babies, CrSO_2_ was 20.6% lower in the first ten minutes of life. For both groups, CrSO_2_ reached a steady state of 70–84% by 10 to 15 min after birth (Table 2).

**Table 2:** Aggregate mean values for cerebral oxygen saturations and cerebral fractional tissue oxygen extraction

	*Time After Birth, minutes*														
	1	2	3	4	5	6	7	8	9	10	11	12	13	14	15
Aggregate CrSO_2_, combined (%)	43.6	42.0	48.0	54.5	61.3	67.6	70.7	73.8	73.9	73.5	76.8	77.2	77.1	77.2	77.3
Aggregate cFTOE, combined	0.37	0.22	0.26	0.21	0.17	0.15	0.15	0.13	0.13	0.14	0.09	0.09	0.09	0.11	0.11
Aggregate CrSO_2_, term (%)	42.9	45.0	48.5	58.2	65.5	68.9	72.2	74.3	75.2	76.5	84.9	84.5	84.4	84.3	83.1
Aggregate cFTOE, term	0.44	0.34	0.29	0.25	0.22	0.19	0.19	0.19	0.18	0.20	0.19	0.19	0.20	0.22	0.23
Aggregate CrSO_2_, preterm (%)	40.4	38.7	44.7	51.8	64.0	67.9	72.2	74.5	76.5	78.0	76.8	78.0	78.2	78.6	78.0
Aggregate cFTOE, preterm	-	0.37	0.33	0.28	0.25	0.23	0.21	0.19	0.18	0.16	0.18	0.18	0.17	0.19	0.19

This suggests a dependant relationship between respiratory adaptations and cerebrovascular functioning within the brain during the neonatal transition period.

There were mixed results on the impact of delivery type on cerebral oxygenation, which may be in part due to the influence of confounders. Most studies included focussed on CrSO_2_ rather than FTOE, making generalisability of FTOE results difficult to interpret. Only five studies focused on extremely preterm infants, so it is unsurprising that there were no significant differences in CrSO_2_/cFTOE values between gestation. Significant differences in cerebral autoregulation are likely to exist and thus further research is needed in extreme preterm infants.

Although there were no significant differences identified between preterm and term babies, this can be explained by selection bias in trial design. This is due to inherent difficulties in defining a ‘normal’ group of preterm babies to be examined: including extremely preterm infants can be challenging due to critical illness and unpredictability as well as the associated ethical implications. Only one study^47^ had an average gestational age of below 26 weeks in this review.

The need for respiratory support was a common exclusion criterion, meaning that only well-adapted preterm babies would be included, giving an inaccurate representation of the true physiological differences in gestation. Moreover, in studies that included preterm infants requiring respiratory support, reference ranges derived from term infants were likely to have been used to regulate oxygen delivery. This has been demonstrated by Wolfsberger et al. in a secondary outcome analysis of the COSGOD III trial,^50^ reporting lower SpO_2_ and CrSO_2_ values in a subgroup of preterm babies who mostly required respiratory support.

There are several limitations to this review. The lack of data on long-term outcomes and cord clamping practices made it difficult to propose recommendations for practice. This has been explored to some extent in the literature through studies which did not meet our inclusion criteria: one ancillary study to the COSGOD pilot trial investigated 31 babies (median age 30^+2^ weeks) demonstrating a significant link between the burden of hypoxia and lower general movement optimality scores (GMOS), indicative of impaired general movements.^69^ Similarly, another retrospective analysis of four studies demonstrated that infants with adverse neurodevelopmental outcomes (defined as either < 70 on a Bayley Scale of Infant Development II/III, significant cognitive impairment or mortality) had lower CrSO_2_ scores during the neonatal transition in comparison to infants with favourable neurodevelopmental outcomes.^51^

As all studies excluded congenital malformations and congenital cardiac disease, it is also difficult to suggest strategies for optimum cord management in these settings. Exact comparison of all extracted data is limited due to the range of machines, algorithms and hardware which were used: INVOS and FORESIGHT devices have differences between up to 10-14%^70^ whilst differences between NIRO and INVOS of 2–12% have also been described.^71^

One study^72^ compared the MASIMO ROOT machine to NIRO and INVOS, demonstrating that although cerebral oxygenation values followed a similar trajectory, CrSO_2_ values from the INVOS device were initially lower but eventually exceeded the ROOT device values.

In this review we found no significant differences between NIRS devices, with an overall good correlation between CrSO_2_, cTOI and cFTOE measurements. The applicability of mean aggregated values is questionable given the theoretical differences in perinatal factors. As most studies focused on healthy term babies born via elective Caesarean section, our results are reflective of the current evidence base.

## Conclusion

NIRS can be feasibly used in the delivery room to guide neonatal stabilisation. Our results show cerebral oxygenation during the transition reliably reaches a steady state by 10–15 minutes of life with no differences identified in term and late preterm infants.

Cerebral regional oxygen saturations are influenced by several other physiological parameters such as pH, pCO_2_, glucose and lactate. Most of the published data is derived from studies in healthy term babies or late preterm babies, accounting for the statistical similarities observed between cohorts. The lack of data on cord clamping practices makes it difficult to suggest optimum cord management strategies. Long-term data is also relatively scarce. Within the studies included, there is evidence to suggest that lower CrSO_2_ values during the transition are associated with intraventricular haemorrhage development,^46,47^ with some correlations to aEEG appearances, although NIRS-derived data from Alderstein et al. suggested that cerebral hyperperfusion may occur prior to IVH development.^73^

NIRS can influence future cord management strategies and enable early identification of patients at risk of adverse neurodevelopmental outcomes. Wider research is needed for preterm infants, congenital malformations, long-term outcomes and high-risk deliveries measuring cerebral NIRS in the settings of delivery room interventions in both observational studies and randomised trials.

## Supplementary information

Supplementary material

## Data Availability

Template data collection forms, data extracted from included studies, and data used for all analyses can be provided upon request from corresponding author.
